# The Role of Hypothermia in Large Hemispheric Infarction: A Systematic Review and Meta-Analysis

**DOI:** 10.3389/fneur.2020.549872

**Published:** 2020-10-27

**Authors:** Jing Li, Yanghui Gu, Gang Li, Lixin Wang, Xiaobin Cheng, Min Wang, Min Zhao

**Affiliations:** ^1^Department of Intensive Care Unit, Hubei Provincial Hospital of Traditional Chinese Medicine, Wuhan, China; ^2^Hubei Province Academy of Traditional Chinese Medicine, Wuhan, China; ^3^Department of Cardiology, Shenzhen Traditional Chinese Medicine Hospital, Shenzhen, China; ^4^Department of Neurology, The Second Affiliated Hospital of Guangzhou University of Chinese Medicine, Guangzhou, China

**Keywords:** hypothermia, infarction, stroke, systematic review, meta-analysis

## Abstract

**Background:** Hypothermia is used in the treatment of large hemispheric infarction (LHI); however, its role in outcomes for LHI patients remains ambiguous. This systematic review and meta-analysis was conducted to evaluate the effect of hypothermia on the outcomes of LHI patients.

**Methods:** We searched MEDLINE, Embase, Cochrane Central Register of Controlled Trials, China Biological Medicine Database, and clinical trials registers before September 21, 2018, and then scanned the reference lists. Randomized controlled trials that compared hypothermia with normothermia in LHI patients were included. Primary outcomes that we reviewed were mortality and neurological outcome. Adverse events during treatment were defined as secondary outcomes. We performed a meta-analysis to calculate pooled risk ratios (RRs), standardized mean differences (SMDs), and 95% confidence intervals (CIs) using fixed-effect models.

**Results:** Three randomized controlled trials involving 131 participants were included. No statistically significant association was revealed between hypothermia and mortality (RR, 1.12; 95% CI, 0.76–1.65). There was significant association between hypothermia and good neurological outcome as assessed by modified Rankin Scale score (mRS of 0–3) of survivors (RR, 2.09; 95% CI, 1.14–3.82), and with neurological outcome by mRS (SMD, −0.54; 95% CI, −1.07 to −0.01). However, significant associations were found between hypothermia and gastrointestinal bleeding, gastric retention, electrolyte derangement, and shivering. No significant differences were detected in the incidence of developing herniation in the rewarming process, pneumonia, cardiac arrhythmia, hemorrhagic transformation, hyperglycemia, hypotension, acute kidney injury, and venous thrombotic events in LHI patients who underwent hypothermia compared with those who had normothermia.

**Conclusions:** This meta-analysis suggested that hypothermia was not associated with mortality in LHI patients. However, it was associated with the improvement of neurological outcome, but with a higher risk of adverse events during treatment. Future studies are needed to demonstrate the efficacy and safety of hypothermia for LHI. The protocol for this systematic review was obtained from PROSPERO (registration number: CRD42018111761).

## Introduction

Large hemispheric infarction (LHI) is commonly associated with arterial occlusion of the proximal middle cerebral artery (MCA) or internal carotid artery (ICA) (1). The incidence of LHI ranges from 10 to 20 per 100,000 people annually, which makes up ~10% of supratentorial ischemic strokes (2). Malignant cerebral edema may follow LHI, which can lead to intracranial hypertension and herniation. Thus, it is also known as malignant MCA infarction (1, 3). Although LHI patients received the strongest medical treatments, the mortality of this life-threatening disease is nearly 80% (1, 3–7). Decompressive hemicraniectomy (DHC), a life-saving treatment, has been demonstrated to reduce mortality (4–9). A meta-analysis of six randomized controlled trials (RCTs) of DHC for LHI showed a reduction in mortality of LHI patient with an increased proportion of severely disabled survivors (10). Moreover, ethical issues should be taken into consideration. Given those limitations, hypothermia may be an optional treatment to LHI patients.

Hypothermia is an effective intervention for neuroprotection available to acute brain injury patients (11). Currently, hypothermia acts as a vital part of routine resuscitation care for cardiac arrest, with substantial evidence supporting its contribution to achieving survival benefit and favorable neurological outcome (12, 13). Regarding ischemic stroke, a meta-analysis examined the efficacy of hypothermia in animal models of cerebral ischemia. The authors of the study noted that hypothermia leads to reduction in infarct volume and improvement in functional outcome compared with normothermia. The target temperature to induce hypothermia varied between 24 and 35°C, with a median of 33°C (14). Additionally, the MCA occlusion rat model of ischemic stroke suggested that a target temperature of 36°C achieved broadly similar benefits to a target temperature of 33°C (15). Apart from these preclinical evidences, some studies indicated that hypothermia is safe and feasible for patients with acute ischemic stroke, regardless of it achieves by endovascular cooling or surface cooling (16, 17). Similarly, hypothermia has been found to be safe and feasible when combined with thrombolysis (18–20). With respect to LHI, the potential effectiveness of hypothermia in improving a favorable clinical outcome for survivors has been noted in many studies (21–23). Specifically, LHI patients seem to benefit more from hypothermia combined with DHC than from hypothermia alone (24). Unfortunately, the adverse events accompanying hypothermia are many, such as pneumonia, hypotension, and shivering, which may limit how often hypothermia is used (21–23, 25). Hypothermia is shown to help reduce the intracranial pressure (ICP) values for LHI patients significantly (23, 25, 26). However, there may be a rebound increase in ICP during or after rewarming, which can likely lead to fatal herniation (25–27), a serious adverse event. Studies have suggested prolonging the rewarming phase that may alleviate the herniation, but the evidence is insufficient (25, 26).

Studies have investigated the efficacy and safety of using hypothermia for LHI patients. Nevertheless, its role in managing the patients suffering from LHI remains debatable (28). Consequently, we performed a meta-analysis of RCTs to evaluate the effects of hypothermia on mortality, neurological outcome, and adverse events in LHI patients compared with normothermia.

## Methods

We followed the Cochrane Handbook for Systematic Reviews of Interventions to conduct this systematic review and meta-analysis (29). We reported the meta-analysis in accordance with the Preferred Reporting Items for Systematic Reviews and Meta-Analyses (PRISMA) statement (30), and we conducted this systematic review adhering to the protocol of PROSPERO in which we registered (registration number: CRD42018111761).

### Eligibility Criteria

The eligibility criteria for this systematic review were as follows. (1) Type of studies: RCTs of hypothermia vs. normothermia. (2) Type of participants: clinical signs of a large supratentorial infarction, infarction involving not <2/3 of the MCA territory confirmed by cranial computed tomography (CT) or magnetic resonance imaging (MRI), with or without the territories of anterior and/or posterior cerebral artery additionally affected (5–7, 31). (3) Type of intervention: hypothermia was defined as a treatment in which the core body temperature was maintained at <36°C, which could be achieved in different ways, including endovascular cooling, surface cooling, or even using pharmacological strategies (15, 31, 32). (4) Type of outcome measures: primary outcomes including mortality and neurological outcome; and secondary outcomes such as adverse events during treatment. (5) No language restrictions, but at minimum, the study should have an abstract in English.

### Search Strategy

The MEDLINE (OvidSP) database, Embase (OvidSP), Cochrane Central Register of Controlled Trials (CENTRAL, OvidSP), China Biological Medicine Database (CBM), and clinical trials registers were searched comprehensively. The final search was conducted on September 21, 2018. Additionally, we searched the reference lists of selected publications, and contacted authors for more information if necessary. Search terms that we used to identify publications included “stroke OR brain ischemia OR cerebrovascular disorders OR ischemia apoplexia OR cerebral occlusion OR cerebral infarction” AND “hypothermia OR cryotherapy OR cooling OR temperature OR normothermia” AND “randomized controlled trial OR randomized OR controlled clinical trial.” The search strategies we used in MEDLINE (OvidSP) are shown in Supplementary Table 1.

### Study Selection and Data Extraction

Two reviewers (JL and YHG) carried out the study selection and data extraction independently throughout the process. In the first sift-prescreening after pretest, two reviewers screened the titles and abstracts independently to identify studies that could be included in the next section. In the second sift-selection after pretest, the two reviewers went through the full texts for studies that met the eligibility criteria. Similar pretests were conducted throughout the process. Next, the two reviewers extracted data from every report using a pre-designed electronic form for data collection. The differences were resolved by discussion, and a third reviewer (LXW) acted as a mediator if an agreement could not be reached. We also established contact with the corresponding authors to get extra information if required.

The data were extracted as follows: (1) first author, publication year, country of origin, and publication type; (2) participant characteristics: sex, age, and baseline National Institutes of Health Stroke Scale (NIHSS) score; (3) type of intervention: the method of inducing hypothermia, target temperature, duration of hypothermia, rewarming rate, whether the patient underwent DHC or did not undergo DHC in the hypothermia group, whether normothermia was maintained, and whether patients underwent DHC in the control group; (4) type of outcome measures: mortality, neurological outcome [assessed by the modified Rankin scale (mRS), Barthel index (BI), or NIHSS], adverse events during treatment (incidence of intracranial hypertension or developing herniation in the rewarming process), adverse events related to hypothermia (such as bleeding events, infections, hyperglycemia, cardiac arrhythmia, hypotension, and shivering), and time points of measurement.

### Quality Assessment

The study quality was assessed using the Cochrane Handbook as a reference for risk of bias (29). It consisted of seven items: random sequence generation (selection bias), allocation concealment (selection bias), blinding of participants and personnel (performance bias), blinding of outcome assessment (detection bias), incomplete outcome data (attrition bias), selective outcome reporting (reporting bias), and other bias. These items were judged as either low risk, high risk, or unclear. Two reviewers (JL and YHG) assessed the risk of bias independently. Inconsistencies were solved by discussion, and a third reviewer (LXW) was involved if they were not resolved.

### Statistical Analysis

For dichotomous outcomes, mortality, neurological outcome of survivors as assessed by mRS (0–3 vs. 4–5), and adverse events during treatment, risk ratios (RRs) and 95% confidence intervals (CIs) were calculated for the meta-analysis. For continuous outcomes and neurological outcome assessed by mRS, standardized mean differences (SMDs) and 95% CI were used. The chi-squared test and *I*^2^ statistic were used to evaluate heterogeneity across the studies. Heterogeneity was considered as acceptable when the *P*-value was >0.1 or the value of *I*^2^ was <50%. If heterogeneity was acceptable, the fixed-effect model was applied to the pooled effect estimate. If not, a sensitivity analysis or subgroup analysis was conducted to explore heterogeneity. A random effects meta-analysis was also conducted. Subgroup analyses were performed based on whether the participants underwent DHC, and on the different measurement times for outcomes. Publication bias was evaluated with a visual inspection of a funnel plot, specifically when there were more than 10 studies included in the meta-analysis (29). Meta-analyses were performed though RevMan V.5.3 software, and a result was considered statistically significant if the *P*-value was <0.05.

## Results

### Study Selection and Characteristics

**Figure 1:**
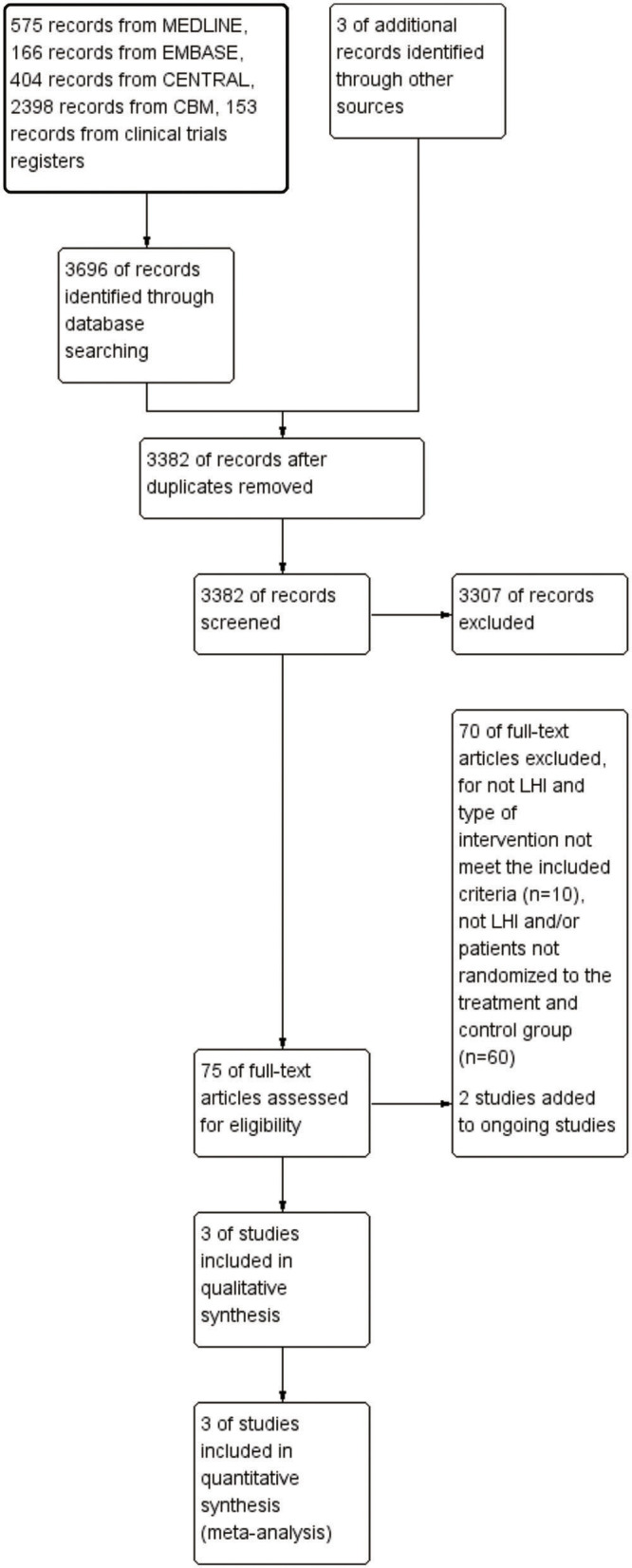
Flow diagram of study selection for the meta-analysis. CENTRAL, Cochrane Central Register of Controlled Trials; CBM, China Biological Medicine Database; LHI, large hemispheric infarction.

There were 3,699 records identified, including 3,696 records from the five databases and three records obtained from searching the reference lists. After removing duplicates, 3,382 records were screened for their titles and abstracts. A total of 75 articles required reading of the full texts, and 70 of them were excluded. Two studies were added to ongoing studies (33, 34). Finally, three studies (21, 24, 35) were included in qualitative synthesis and meta-analysis (Figure 1).

Three RCTs published between 2006 and 2018 with 131 participants were included. Two studies were conducted in China, while one was conducted in Germany. All of the three studies had reported the age, gender, and baseline NIHSS of the two groups, and two of these studies had elaborated on patients' underlying diseases. For baseline of the three studies, no statistical significance was found between the hypothermia group and the control group, apart from one study in which age and gender were not comparable (21). All of the three studies had described at length the phases of induction, maintenance, and rewarming for hypothermia, and normothermia was maintained in the control groups. Mortality was measured within the first week after onset of LHI, and at 3 and 6 months of follow-up. Neurological outcome was assessed by mRS, which was expressed as mean ± standard deviation (SD) in one study, while in the other two studies, ranking from 0 to 3 was defined as a good neurological outcome for survivors. Out of the two studies, one study listed the mRS for each participant included, which could be used for calculating mean and SD. Adverse events were observed during treatment in three studies. Characteristics of the three studies are shown in Table 1.

**Table 1:** Characteristics of the included studies and participants.

**References**	**Country**	**Women/n**		**Age, years (mean** **±** **SD)**		**Baseline NIHSS (mean** **±** **SD)**		**Intervention**							**Outcomes and time points**	
							**HG**					**CG**				
		**HG**	**CG**	**HG**	**CG**	**HG**	**CG**	**Induced hypothermia**	**Target temperature**	**Duration of hypothermia**	**Rewarming rate**	**DHC**	**Temperature**	**DHC**	**Mortality**	**Neurological outcome**
Els et al. (24) 2006	Germany	6/12	4/13	49 ± 12	49 ± 6	18 ± 2	19 ± 2	10 patients via endovascular cooling; 2 patients via surface cooling	35°C	48 h	1°C/24 h	Underwent	Maintained normothermia	Underwent	Within the first week after the onset	At 6 months of follow-up
Su et al. (21) 2016	China	1/16	7/17	59.8 ± 8.6	68.5 ± 8.5	19.7 ± 2.9	20.4 ± 3.8	Endovascular cooling	33 or 34°C	24–72 h	Raised 0.5°C every 12 h; rewarming rate was 0.1°C/h	Did not undergo	Maintained normothermia	Did not undergo	At 6 months of follow-up	At 6 months of follow-up
Liang et al. (35) 2018	China	7/37	11/36	61.2 ± 6.2	60.6 ± 5.8	19.6 ± 2.4	19.7 ± 2.6	Surface cooling	32–35°C	5–7 days	Duration of more than 8 h from 34 to 36°C; rewarming phase, more than 12 h	Did not undergo	Maintained normothermia	Did not undergo	At 3 months of follow-up	At 3 months of follow-up

*HG, hypothermia group; CG, control group; SD, standard deviation; NIHSS, National Institutes of Health Stroke Scale score; DHC, decompressive craniectomy; RCT, randomized controlled trial*.

### Risk of Bias Within Studies and Publication Bias

The seven items for risk of bias are shown graphically (Supplementary Figure 1) and summarized (Supplementary Figure 2) in supplemental data. Both performance bias and detection bias were assessed separately based on whether the outcomes were subjective or objective. Lack of blinding for mortality was unlikely to influence performance; therefore, we judged this factor as low risk of bias for all studies. For detection bias, lack of blinding for mortality and adverse events were unlikely to influence the outcome assessment; thus, we judged these factors as low risk of bias for all studies. Publication bias could not be assessed by examining a funnel plot because of the limited number of included studies.

### Primary Outcomes

#### Mortality

**Figure 2:**
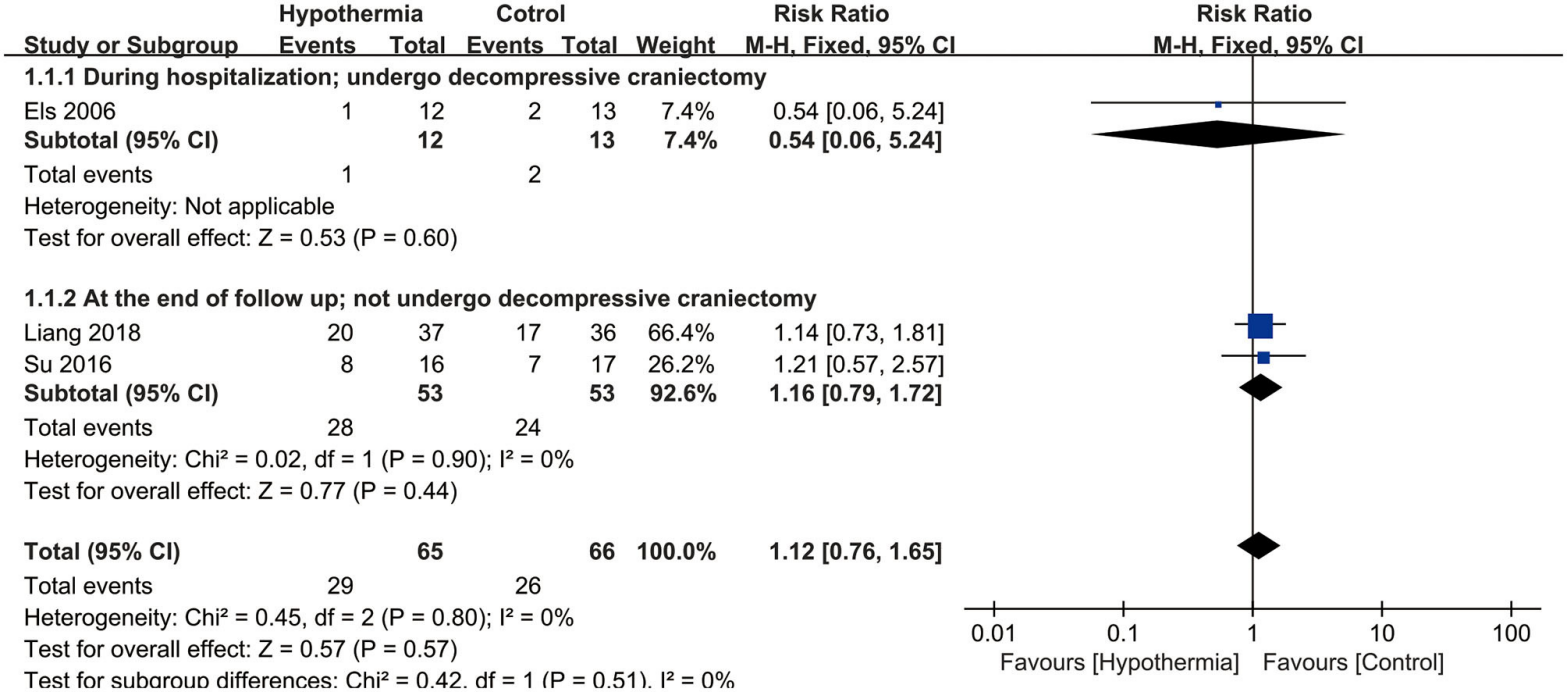
Meta-analysis of association between hypothermia and the mortality of large hemispheric infarction (LHI).

All of the three studies reported mortality (*n* = 131). Our analysis did not find a statistically significant association between hypothermia and mortality when compared with subjects who maintained normothermia (RR, 1.12; 95% CI, 0.76–1.65). There was no heterogeneity observed across studies (*P* = 0.80; *I*^2^ = 0%). The results of meta-analysis are presented in Figure 2.

#### Neurological Outcome

**Figure 3:**
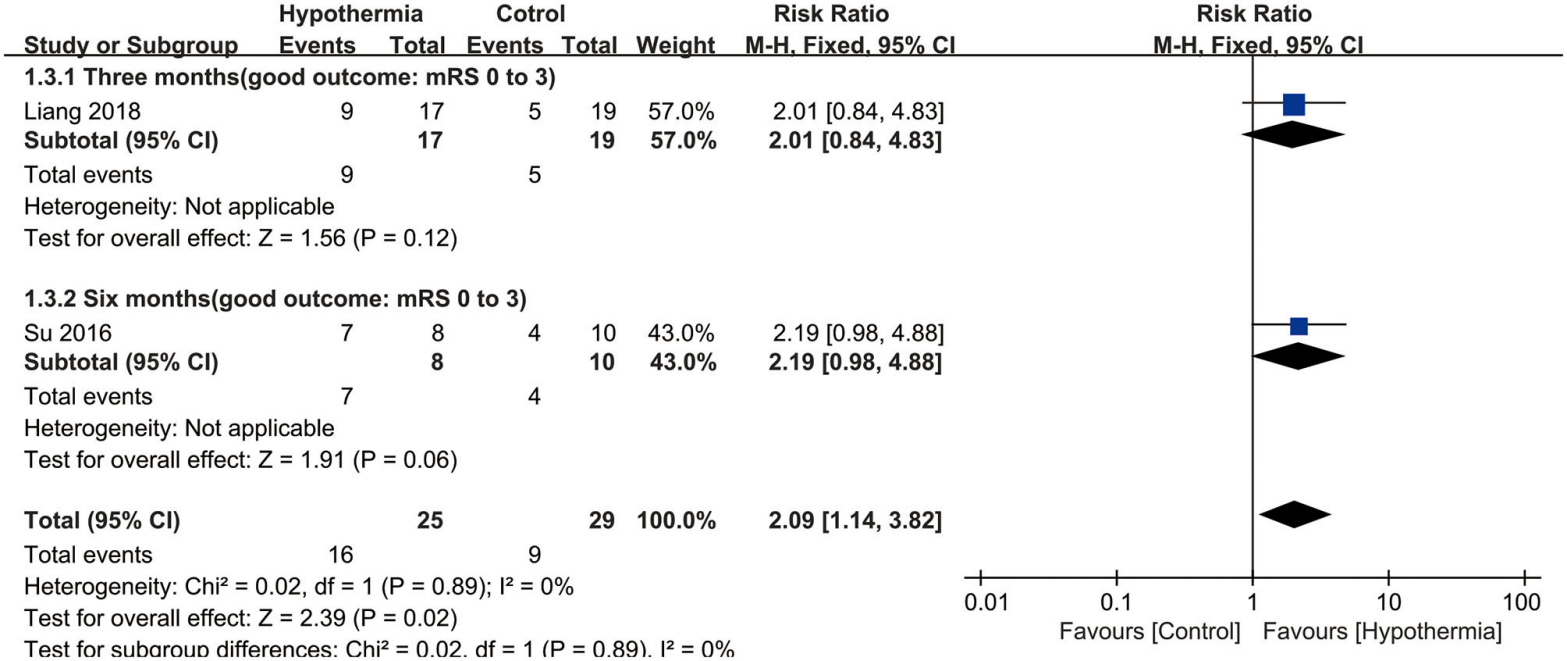
Meta-analysis of association between hypothermia and a good neurological outcome for survivors.

**Figure 4:**
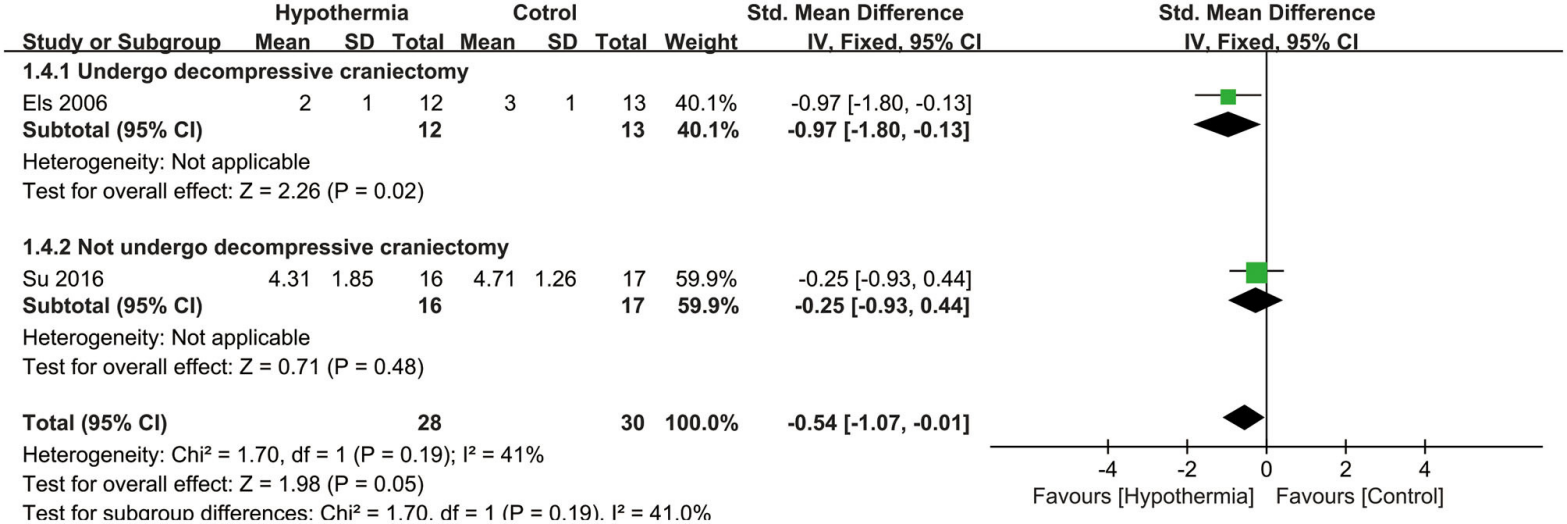
Meta-analysis of association between hypothermia and a low level of mRS for large hemispheric infarction (LHI).

Three studies reported neurological outcome as assessed by mRS. Two of them presented it as a dichotomous variable of good neurological outcome for survivors (*n* = 54 participants). As seen in Figure 3, hypothermia was associated with a significantly higher incidence of good neurological outcome for survivors than for subjects who maintained normothermia (RR, 2.09; 95% CI, 1.14–3.82). No heterogeneity was found between these studies (*P* = 0.89; *I*^2^ = 0%). The values were expressed as mean ± SD of mRS in two studies (*n* = 58 participants). As shown in Figure 4, we found a significant association between hypothermia and a lower level of mRS compared with subjects who maintained normothermia (SMD, −0.54; 95% CI, −1.07 to −0.01). It showed low heterogeneity between the two studies (*P* = 0.19; *I*^2^ = 41%).

### Secondary Outcomes

#### Incidence of Intracranial Hypertension or Developing Herniation in Rewarming Process

Only one study with 73 participants reported data in which herniation developed in the rewarming process. Herniation during the rewarming process following hypothermia was not significantly higher than after normothermia (RR, 1.46; 95% CI, 0.45–4.74). No study had details for incidence of intracranial hypertension.

#### Adverse Events Related to Hypothermia

All of the three studies reported the adverse events related to hypothermia, while one study had no details for the hypothermia group or the control group, and thus was excluded. In total, 106 participants were included in the analysis. There were significantly higher risks of gastrointestinal bleeding (RR, 3.24; 95% CI, 1.62–6.49), gastric retention (RR, 2.43; 95% CI, 1.47–3.99), and electrolyte derangement (RR, 1.59; 95% CI, 1.12–2.28), following hypothermia compared with following normothermia. There were no significant differences found in the association between hypothermia and incidences of pneumonia (RR, 1.40; 95% CI, 0.87–2.25), cardiac arrhythmia (RR, 1.20; 95% CI, 0.75–1.91), hemorrhagic transformation (RR, 0.67; 95% CI, 0.20–2.23), hyperglycemia (RR, 1.36; 95% CI, 0.68–2.69), hypotension (RR, 1.87; 95% CI, 0.76–4.60), and acute kidney injury (RR, 5.00; 95% CI, 0.90–27.71) compared with normothermia. Significant associations were found between hypothermia and shivering (RR, 5.84; 95% CI, 1.88–18.12) in one study with 73 participants. Only one study with 33 participants looked at venous thrombotic events, and no significant associations were found (RR, 2.13; 95% CI, 0.45–10.05). These findings are shown in Supplementary Figure 3.

There was low heterogeneity detected between studies for adverse events related to hypothermia, with the exception of substantial heterogeneity for hyperglycemia (*P* = 0.06; *I*^2^ = 72%). A random effects model was applied for hyperglycemia (RR, 1.89; 95% CI, 0.28–12.57); however, it did not change the result much (*P* = 0.07; *I*^2^ = 70%) (Supplementary Figure 4). As described in Cochrane Handbook, it is questionable to investigate heterogeneity when the involved studies are few. We chose not to perform meta-analysis for hyperglycemia, as the effect estimate was pooled from only two studies. The final results are shown in Table 2.

**Table 2:** The results of meta-analysis for adverse events during treatment.

**Group/subgroup**	**No. of studies**	**Adverse events/n**		**Test for heterogeneity**		**Test for all effect**		**Analysis model**	**Meta-analysis**	
		**HG**	**CG**	***I*^2^**	***P***	***Z***	***P***		**Risk ratio**	**95% CI**
Developed herniation in the rewarming process	1	6**/**37	4/36	–*	–	0.63	0.53	Fixed effect	1.46	(0.45, 4.74)
Pneumonia	2	25/53	18/53	39%	0.20	1.37	0.17	Fixed effect	1.40	(0.87, 2.25)
Cardiac arrhythmia	2	22/53	18/53	33%	0.22	0.77	0.44	Fixed effect	1.20	(0.75,1.91)
Hemorrhagic transformation	2	4/53	6/53	0%	0.82	0.66	0.51	Fixed effect	0.67	(0.20, 2.23)
Gastrointestinal bleeding	2	26/53	8/53	0%	0.65	3.32	0.0009	Fixed effect	3.24	(1.62, 6.49)
Gastric retention	2	31/53	13/53	0%	0.79	3.49	0.0005	Fixed effect	2.43	(1.47, 3.99)
Hyperglycemia	2	15/53	11/53	72%	0.06	0.87	0.38	Fixed effect	1.36	(0.68, 2.69)
Hypotension	2	11/53	6/53	0%	0.77	1.36	0.17	Fixed effect	1.87	(0.76, 4.60)
Electrolyte derangement	2	35/53	22/53	0%	0.45	2.56	0.01	Fixed effect	1.59	(1.12, 2.28)
Shivering	1	18/37	3/36	–	–	3.05	0.002	Fixed effect	5.84	(1.88, 18.12)
Acute kidney injury	2	7/53	1/53	0%	0.96	1.84	0.07	Fixed effect	5.00	(0.90, 27.71)
Venous thrombotic events	1	4/16	2/17	–	–	0.95	0.34	Fixed effect	2.13	(0.45, 10.05)

*HG, hypothermia group; CG, control group*.

* *Only one study was included and heterogeneity test was not done*.

### Subgroup Analyses

For mortality, subgroup analyses were performed according to whether the participants underwent DHC or did not undergo it, and at different measurement time points during hospitalization or at the end of follow up. Neither participants with DHC nor without DHC showed a statistically significant association with mortality (RR: 0.54, 95% CI, 0.06–5.24; RR: 1.16, 95% CI, 0.79–1.72, respectively). No significant associations were found between hypothermia and mortality compared with normothermia, neither during hospitalization nor at the end of follow-up (RR: 0.54, 95% CI, 0.06–5.24; RR: 1.16, 95% CI, 0.79–1.72, respectively). Both subgroup analyses are shown in Figure 2. For neurological outcome as well, subgroup analyses were conducted according to whether the participants underwent DHC or did not undergo it, and at measurement time of 3 or 6 months of follow-up. For patients who underwent DHC, a statistically significant association between hypothermia and neurological outcome was observed compared with normothermia (RR, −0.97; 95% CI, −1.80 to −0.13). Comparing hypothermia with normothermia, the time at which outcomes were measured did not significantly affect the results (Figures 3, 4).

## Discussion

This systematic review included three RCTs to evaluate the effects of hypothermia on mortality, neurological outcome, and adverse events. LHI is associated with high mortality and disability rate. The major reasons for it could be the brain swelling that occurs within 1–5 days after the onset of LHI, intracranial hypertension, or even herniation (1, 2). As confirmed in a prior meta-analysis, fever after stroke insult is highly related to an increase in mortality and poor neurological outcome (36). In this way, acute stroke patients might benefit from hypothermia. Hypothermia may have multiple mechanisms of action on ischemic stroke; for instance, it could preserve the neuronal integrity and improve their chance of survival, decrease brain metabolism, reduce the production of free radicals and depress the reactions of inflammation, attenuate apoptosis, stabilize the blood–brain barrier, and decrease the cytotoxic edema (2, 28, 37). There may be other pathways involved as well. These benefits contribute to mitigating brain swelling, reducing ICP, and providing neuroprotection.

Our findings showed that hypothermia was not significantly associated with a reduction in mortality of LHI patients. Similarly, we found that neither a previous meta-analysis with six RCTs involving 252 patients who suffered from acute ischemic stroke (38) nor another meta-analysis including eight trials with 423 patients of acute stroke (39) detected a significant reduction in mortality following hypothermia. The correlative factors accounting for mortality in LHI patients were complex and diverse, and that includes limited sample size, which might have resulted in this finding. The potential correlation between hypothermia and mortality needs to be further investigated.

In our review, neurological outcome was assessed by the mRS, which is widely used to measure neurological outcome for stroke patients in RCTs. The pooled estimate showed that there was statistically significant association between hypothermia and good neurological outcome for survivors. Additionally, there was significant association of hypothermia with improvement in neurological outcome. These positive effects are likely due to the neuroprotection of hypothermia. Our findings were consistent with several clinical studies conducted on LHI patients (21–24, 40). Some of these were case series reports or case control study that provided insufficient evidence for hypothermia (22, 23, 40). The other RCTs only found a trend of hypothermia leading to good neurological outcome, with conclusions drawn cautiously (21, 24). The sample sizes of these individual studies were so small that no effects were detected, but it was likely that we would enhance the estimation of treatment effect when we combined these studies. However, in a prior meta-analysis for acute ischemic stroke, no significant association was revealed between hypothermia and neurologic outcome (38). The different definitions of neurological outcome and the inclusion of mild or moderate stroke patients might explain this difference between the results in the different studies.

We found that hypothermia was a double-edged sword; it had some benefits in the neurological outcome of LHI patients, but there was an increase in adverse events during treatment as well. The most serious adverse event we noted was the incidence of intracranial hypertension or herniation developing in the rewarming process, which likely results in death of the patient. A rebound increase in ICP during or after rewarming had been observed in several studies (25–27). Prolonging the rewarming phase might be conducive to controlling this unfavorable situation (25, 26). However, the rewarming duration (<12 or ≥12 h) had no significant effect on mortality or neurological outcome for acute ischemic stroke in a preceding meta-analysis (38). In our meta-analysis, we found that LHI patients did not likely risk a higher incidence of developing herniation in the rewarming process, but the evidence was insufficient as only one study examined this. There were no studies that reported intracranial hypertension following hypothermia in these patients. Further research is needed to clarify this issue. However, we found that hypothermia was significantly associated with a higher risk of gastrointestinal bleeding, gastric retention, electrolyte derangement, and shivering. No significant differences were detected between hypothermia and normothermia in relation to subsequent pneumonia, cardiac arrhythmia, hemorrhagic transformation, hyperglycemia, hypotension, acute kidney injury, and venous thrombotic events. A meta-analysis by Wan et al. (38) found a significant difference between hypothermia and standard treatment in the incidence of pneumonia for acute ischemic stroke. However, the conditions of the patients in our study were more severe than those in the study by Wan et al., which might account for this inconsistency. The patients in our study were usually more likely to be dysphagic and unconscious, which gave rise to pneumonia (41). Generally, the common adverse events related to hypothermia were not fatal, including pneumonia, bradycardia, hypotension, shivering, and coagulopathy (21–23, 25). Although these adverse events could be managed, the safety of hypothermia remained to be evaluated for LHI. EuroHYP-1 (European multicenter, randomized, phase III clinical trial) was conducted to assess whether hypothermia could improve functional outcome at 3 months for awake acute ischemic stroke patients. The enrollment was halted after 98 patients instead of the original 6-year target of 1,500 patients in 36 trial sites. Furthermore, just one-third of patients who were randomized to receive hypothermia had achieved the cooling target. The most important reason for not reaching the predefined target was shivering (42). Hypothermia may be more operable in LHI patients than in awake acute ischemic stroke patients, as most LHI patients are not conscious. Because of these difficulties, such studies are challenging to perform. There was low heterogeneity detected between studies for all outcomes related to hypothermia, except for hyperglycemia. The varied definition of hyperglycemia, with different levels of blood glucose, may be the source of this heterogeneity.

Subgroup analyses were conducted based on patients who underwent DHC or did not undergo it and at different measurement times. For patients who underwent DHC, statistical significance was detected between hypothermia and normothermia in terms of neurological outcome. A study has shown that LHI patients might achieve more advantages from combining hypothermia with DHC compared with DHC alone (24). DHC has been widely used for LHI patients to decrease ICP directly. When comparing hypothermia with DHC, similar maximal ICP values were found in the two groups, and no statistical significance was reached in the increased incidence of ICP beyond 20 mmHg (27). The combination of hypothermia and DHC has been promising. Two RCTs about this approach are under way, including Decompressive Craniectomy Combined with Mild Hypothermia in Patients with Large Hemispheric Infarction (ChiCTR-TRC-12002698) (33) and Decompressive Surgery Plus Hypothermia for Space-Occupying Stroke (DEPTH-SOS, DRKS00000623) (34). As reported, it took 5–6 months after stroke onset for spontaneous recovery of neurological outcome to reach a plateau, particularly in more severe stroke cases (43). However, there was no statistical significance for different measurement time in our review, which might be due to the small sample size.

We note several strengths in our study. First, our review was conducted strictly in accordance with the Cochrane Handbook; our analytic plan was pre-specified in protocol with a rigorous and scientific methodology. Second, our literature search was comprehensive, with neither language restrictions nor publication status limitations. After searching comprehensively, only three RCTs were included in our review. We found that the small sample size was an inherent limitation in this type of clinical trial due to the complexity and variability of LHI patients, clinical studies were too difficult to carry out. Third, the three RCTs were assessed to have a low risk of bias in general, which increased the reliability of the results. Fourth, we conducted subgroup analyses on participants who underwent DHC or did not undergo it, which might act as an alert to the possible advantages of combining hypothermia and DHC for the management of LHI. Finally, we found that hypothermia might contribute to improving neurological outcome after pooling effect estimates, a result which was not obvious in individual studies. To conclude, this is the first systematic review and meta-analysis performed to investigate the effect of hypothermia on patients with LHI.

Several limitations in our study merit careful consideration. The main shortcomings are the very few studies and the small sample size of patients included in our meta-analysis. Unfortunately, the strength and quality of evidence might be restricted by this paucity, which ought to be taken cautiously in clinical practice. However, a small number of studies does not mean the results are meaningless. Many systematic reviews from the Cochrane Library include less than three studies, or even none, but they are still considered clinically important (44–47). Although the number of studies was small, we hope to draw attention to the role of hypothermia in LHI. Regarding adverse events, only two studies had reported these in detail, and the safety of hypothermia should be further evaluated. Also, we failed to obtain the mRS score of each patient at 6 months after stroke onset from the study (24), so two meta-analyses were performed on neurological outcome assessed by mRS as dichotomous outcome and continuous outcome. Finally, the definition of normothermia ranges from 36 to 37.8°C in different studies (48, 49), however a target temperature <36°C is considered as hypothermia (31), it may affect the results.

## Conclusions

This meta-analysis suggested that hypothermia was not associated with the mortality of LHI patients, but it was associated with the improvement of neurological outcome. Meanwhile, we found that hypothermia was associated with higher risk of gastrointestinal bleeding, gastric retention, electrolyte derangement, and shivering. It was, however, not associated with the risk of developing herniation in the rewarming process, pneumonia, cardiac arrhythmia, hemorrhagic transformation, hyperglycemia, hypotension, acute kidney injury, and venous thrombotic events. Given the limited number of studies and low sample size, the evidence should be treated cautiously when applied to clinical practice. Future studies are needed to demonstrate the efficacy and safety of hypothermia for LHI patients.

## Author Contributions

GL and LW contributed to the conception, design, and data interpretation of this study. JL and YG contributed to the study selection, data extraction, quality assessment, and data analysis. JL drafted the manuscript. XC and MW designed the search strategy and performed the search. XC, MW, and MZ completed the tables and figures. MZ critically revised the manuscript. All authors read and approved the final manuscript.

## Conflict of Interest

The authors declare that the research was conducted in the absence of any commercial or financial relationships that could be construed as a potential conflict of interest.

## References

[1] Hacke W Schwab S Horn M Spranger M de Georgia M von Kummer R 'Malignant' middle cerebral artery territory infarction: clinical course and prognostic signs. Arch Neurol. (1996) 53:309–15. 8929152 10.1001/archneur.1996.00550040037012

[2] Huttner HB Schwab S. Malignant middle cerebral artery infarction: clinical characteristics, treatment strategies, and future perspectives. Lancet Neurol. (2009) 8:949–58. 10.1016/S1474-4422(09)70224-8 19747656

[3] Berrouschot J Sterker M Bettin S Koster J Schneider D Mortality of space-occupying ('malignant') middle cerebral artery infarction under conservative intensive care. Intensive Care Med. (1998) 24:620–3. 10.1007/s001340050625 9681786

[4] Vahedi K Vicaut E Mateo J Kurtz A Orabi M Guichard J. Sequential-design, multicenter, randomized, controlled trial of early decompressive craniectomy in malignant middle cerebral artery infarction (DECIMAL trial). Stroke. (2007) 38:2506–17. 10.1161/STROKEAHA.107.485235 17690311

[5] Jüttler E Unterberg A Woitzik J Bösel J Amiri H Sakowitz OW. Hemicraniectomy in older patients with extensive middle-cerebral-artery stroke. N Engl J Med. (2014) 370:1091–100. 10.1056/NEJMoa1311367 24645942

[6] Jüttler E Schwab S Schmiedek P Unterberg A Hennerici M Woitzik J. Decompressive surgery for the treatment of malignant infarction of the middle cerebral artery (DESTINY). Stroke. (2007) 38:2518–25. 10.1161/STROKEAHA.107.485649 17690310

[7] Hofmeijer J Kappelle LJ Algra A Amelink GJ van Gijn J van der Worp HB. Surgical decompression for space-occupying cerebral infarction (the Hemicraniectomy after middle cerebral artery infarction with life-threatening edema trial [HAMLET]): a multicentre, open, randomised trial. Lancet Neurol. (2009) 8:326–33. 10.1016/S1474-4422(09)70047-X 19269254

[8] Zhao J Su Y Zhang Y Zhang Y Zhao R Wang L. Decompressive hemicraniectomy in malignant middle cerebral artery infarct: a randomized controlled trial enrolling patients up to 80 years old. Neurocrit Care. (2012) 17:161–71. 10.1007/s12028-012-9703-3 22528280

[9] Frank JI Schumm LP Wroblewski K Chyatte D Rosengart AJ Kordeck C. Hemicraniectomy and durotomy upon deterioration from infarction-related swelling trial. Stroke. (2014) 45:781–7. 10.1161/STROKEAHA.113.003200 24425122 PMC4033520

[10] Back L Nagaraja V Kapur A Eslick GD. Role of decompressive hemicraniectomy in extensive middle cerebral artery strokes: a meta-analysis of randomised trials. Intern Med J. (2015) 45:711–7. 10.1111/imj.12724 25684396

[11] Choi HA Badjatia N Mayer SA. Hypothermia for acute brain injury-mechanisms and practical aspects. Nat Rev Neurol. (2012) 8:214–2. 10.1038/nrneurol.2012.21 22371279

[12] Arrich J Holzer M Havel C Müllner M Herkner H. Hypothermia for neuroprotection in adults after cardiopulmonary resuscitation. Cochrane Database Syst Rev. (2016) 2:CD004128. 10.1002/14651858.CD004128.pub4 26878327 PMC6516972

[13] Schenone AL Cohen A Patarroyo G Harper L Wang X Shishehbor MH. Therapeutic hypothermia after cardiac arrest: a systematic review/meta-analysis exploring the impact of expanded criteria and targeted temperature. Resuscitation. (2016) 108:102–10. 10.1016/j.resuscitation.2016.07.238 27521472

[14] van der Worp HB Sena ES Donnan GA Howells DW Macleod MR. Hypothermia in animal models of acute ischaemic stroke: a systematic review and meta-analysis. Brain. (2007) 130:3063–74. 10.1093/brain/awm083 17478443

[15] Lee JH Lim J Chung YE Chung SP Park I Kim CH. Targeted temperature management at 33°C or 36°C produces equivalent neuroprotective effects in the middle cerebral artery occlusion rat model of ischemic stroke. Shock. (2018) 50:714–9. 10.1097/SHK.0000000000001106 29337840

[16] Geurts M Petersson J Brizzi M Olsson-Hau S Luijckx G Algra A. COOLIST (Cooling for ischemic stroke trial). Stroke. (2017) 48:219–21. 10.1161/STROKEAHA.116.014757 27856954

[17] de Georgia MA Krieger DW Abou-Chebl A Devlin TG Jauss M Davis SM. Cooling for acute ischemic brain damage (COOL AID): a feasibility trial of endovascular cooling. Neurology. (2004) 63:312–7. 10.1212/01.WNL.0000129840.66938.75 15277626

[18] Hemmen TM Raman R Guluma KZ Meyer BC Gomes JA Cruz-Flores S. Intravenous thrombolysis plus hypothermia for acute treatment of ischemic stroke (ICTuS-L). Stroke. (2010) 41:2265–70. 10.1161/STROKEAHA.110.592295 20724711 PMC2947593

[19] Piironen K Tiainen M Mustanoja S Kaukonen KM Meretoja A Tatlisumak T. Mild hypothermia after intravenous thrombolysis in patients with acute stroke: a randomized controlled trial. Stroke. (2014) 45:486–91. 10.1161/STROKEAHA.113.003180 24436240

[20] Ma L Song L Yu X Yu T Liang H Qiu J The clinical study on the treatment for acute cerebral infarction by intra-arterial thrombolysis combined with mild hypothermia. Eur Rev Med Pharmacol Sci. (2017) 21:1999–2006. 28485774

[21] Su Y Fan L Zhang Y Zhang Y Ye H Gao D. Improved neurological outcome with mild hypothermia in surviving patients with massive cerebral hemispheric infarction. Stroke. (2016) 47:457–63. 10.1161/STROKEAHA.115.009789 26696645

[22] Milhaud D Thouvenot E Heroum C Escuret E. Prolonged moderate hypothermia in massive hemispheric infarction: clinical experience. J Neurosurg Anesthesiol. (2005) 17:49–53. 10.1002/jbm.a.10551 15632543

[23] Schwab S Schwarz S Spranger M Keller E Bertram M Hacke W. Moderate hypothermia in the treatment of patients with severe middle cerebral artery infarction. Stroke. (1998) 29:2461–6. 10.1161/01.STR.29.12.2461 9836751

[24] Els T Oehm E Voigt S Klisch J Hetzel A Kassubek J. Safety and therapeutical benefit of hemicraniectomy combined with mild hypothermia in comparison with hemicraniectomy alone in patients with malignant ischemic stroke. Cerebrovasc Dis. (2006) 21:79–85. 10.1159/000090007 16330868

[25] Schwab S Georgiadis D Berrouschot J Schellinger PD Graffagnino C Mayer SA. Feasibility and safety of moderate hypothermia after massive hemispheric infarction. Stroke. (2001) 32:2033–5. 10.1161/hs0901.095394 11546893

[26] Steiner T Friede T Aschoff A Schellinger PD Schwab S Hacke W. Effect and feasibility of controlled rewarming after moderate hypothermia in stroke patients with malignant infarction of the middle cerebral artery. Stroke. (2001) 32:2833–5. 10.1161/hs1201.99511 11739982

[27] Georgiadis D Schwarz S Aschoff A Schwab S. Hemicraniectomy and moderate hypothermia in patients with severe ischemic stroke. Stroke. (2002) 33:1584–8. 10.1161/01.STR.0000016970.51004.D9 12052995

[28] Tahir RA Pabaney AH. Therapeutic hypothermia and ischemic stroke: a literature review. Surg Neurol Int. (2016) 7:S381–6. 10.4103/2152-7806.183492 27313963 PMC4901811

[29] Higgins JP Green S Cochrane Handbook for Systematic Reviews of Interventions: Cochrane Book Series. Chichester: John Wiley & Sons Ltd (2011).

[30] Moher D Liberati A Tetzlaff J Altman DG Preferred reporting items for systematic reviews and meta-analyses: the PRISMA statement. BMJ. (2009) 339:b2535 10.1136/bmj.b2535 19622551 PMC2714657

[31] Torbey MT Bosel J Rhoney DH Rincon F Staykov D Amar AP. Evidence-based guidelines for the management of large hemispheric infarction: a statement for health care professionals from the neurocritical care society and the German society for neuro-intensive care and emergency medicine. Neurocrit Care. (2015) 22:146–64. 10.1007/s12028-014-0085-6 25605626

[32] Lewis SR Evans DJW Butler AR Schofield-Robinson OJ Alderson P. Hypothermia for traumatic brain injury (review). Cochrane Database Syst Rev. (2017) 9:CD001048. 10.1002/14651858.CD001048.pub5 28933514 PMC6483736

[33] Su Y Fan L Ding Y Sung G Zhang Y Gao D Decompressive craniectomy combined with mild hypothermia in patients with large hemispheric infarction - a randomised controlled trial. In: 27th European Stroke Conference 2018-04-11 (Athens) (2018). 10.1186/s12883-021-02142-7 PMC7953537 33711963

[34] Neugebauer H Kollmar R Niesen WD Bosel J Schneider H Hobohm C. DEcompressive surgery Plus hypoTHermia for space-occupying stroke (DEPTH-SOS): a protocol of a multicenter randomized controlled clinical trial and a literature review. Intern J Stroke. (2013) 8:383–7. 10.1111/ijs.12086 23782729

[35] Liang K Zhao J Yao Y Jiang X Chen Y Therapeutic hypothermia in patients with acute large hemispheric infarction:a randomized controlled clinical trial. Chin J Neuro. (2018) 51:34–8. 10.3760/cma.j.issn.1006-7876.2018.01.007

[36] Hajat C Hajat S Sharma P. Effects of poststroke pyrexia on stroke outcome: a meta-analysis of studies in patients. Stroke. (2000) 31:410–4. 10.1161/01.STR.31.2.410 10657414

[37] Gonzalez-Ibarra FP Varon J Lopez-Meza EG Therapeutic hypothermia: critical review of the molecular mechanisms of action. Front Neurol. (2011) 2:4 10.3389/fneur.2011.00004 21331282 PMC3035015

[38] Wan Y Nie C Wang H Huang C. Therapeutic hypothermia (different depths, durations, and rewarming speeds) for acute ischemic stroke: a meta-analysis. J Stroke Cerebrovasc Dis. (2014) 23:2736–47. 10.1016/j.jstrokecerebrovasdis.2014.06.017 25238926

[39] Den Hertog HM van der Worp HB Tseng MC Dippel DW. Cooling therapy for acute stroke (Review). Cochrane Database Syst Rev. (2009) 1:CD001247. 10.1002/14651858.CD001247.pub2 19160194 PMC7073930

[40] Schwab S Schwarz S Aschoff A Keller E Hacke W. Moderate hypothermia and brain temperature in patients with severe middle cerebral artery infarction. Acta Neurochir Suppl. (1998) 71:131–4. 10.1007/978-3-7091-6475-4_39 9779165

[41] Martino R Foley N Bhogal S Diamant N Speechley M Teasell R. Dysphagia after stroke: incidence, diagnosis, and pulmonary complications. Stroke. (2005) 36:2756–63. 10.1161/01.STR.0000190056.76543.eb 16269630

[42] van der Worp HB Macleod MR Bath PM Bathula R Christensen H Colam B. Therapeutic hypothermia for acute ischaemic stroke. Results of a European multicentre, randomised, phase III clinical trial. Eur Stroke J. (2019) 4:254–62. 10.1177/2396987319844690 31984233 PMC6960691

[43] Duncan PW Jorgensen HS Wade DT. Outcome measures in acute stroke trials: a systematic review and some recommendations to improve practice. Stroke. (2000) 31:1429–38. 10.1161/01.STR.31.6.1429 10835468

[44] Lin S Liu M Wu B Hao Z Yang J Tao W. External counterpulsation for acute ischaemic stroke. Cochrane Database Syst Rev. (2012) 1:CD009264. 10.1002/14651858.CD009264.pub2 22259001 PMC11500817

[45] Li LR Chao Y Chaudhary B. Intraoperative mild hypothermia for postoperative neurological deficits in people with intracranial aneurysm. Cochrane Database Syst Rev. (2016) 3:CD008445. 10.1002/14651858.CD008445.pub3 27000210 PMC6599874

[46] Bereczki D Fekete I Prado GF Liu M. Mannitol for acute stroke. Cochrane Database Syst Rev. (2007) 2007:CD001153. 10.1002/14651858.CD001153.pub2 17636655 PMC7032636

[47] Saxena M Andrews PJ Cheng A Deol K Hammond N. Modest cooling therapies (35°C to 37.5°C) for traumatic brain injury. Cochrane Database Syst Rev. (2014) 2014:CD006811. 10.1002/14651858.CD006811.pub3 25135381 PMC7389311

[48] Mattsson N Zetterberg H Nielsen N Blennow K Dankiewicz J Friberg H. Serum tau and neurological outcome in cardiac arrest. Ann Neurol. (2017) 82:665–75. 10.1002/ana.25067 28981963 PMC5725735

[49] Lilja G Nielsen N Ullen S Blennow NE Dankiewicz J Friberg H. Protocol for outcome reporting and follow-up in the targeted hypothermia versus targeted normothermia after out-of-hospital cardiac arrest trial (TTM2). Resuscitation. (2020) 150:104–12. 10.1016/j.resuscitation.2020.03.004 32205155

