# Supplementary material for: The Role of Hypothermia in Large Hemispheric Infarction: A Systematic Review and Meta-Analysis
Source: Front Neurol. 2020 Oct 27;11:549872. doi: 10.3389/fneur.2020.549872 (PMC7653189; doi:10.3389/fneur.2020.549872)
Supplement: Supplementary file 1 [file Data_Sheet_1.PDF]

SUPPLEMENTAL MATERIAL

Supplementary Table

Table S1: Search strategy for MEDLINE (OvidSP)

Search strategy for MEDLINE (OvidSP)
1. cerebrovascular disorders/ or basal ganglia cerebrovascular disease/ or exp brain ischemia/ or carotid artery diseases/ or carotid artery thrombosis/ or intracranial arterial diseases/ or cerebral arterial diseases/ or exp "intracranial embolism and thrombosis"/ or exp stroke/
2. (isch?emi$ adj6 (stroke$ or apoplex$ or cerebral vasc$ or cerebrovasc$ or cva or attack$)).tw.
3. ((brain or cerebr$ or cerebell$ or vertebrobasil$ or hemispher$ or intracran$ or intracerebral or infratentorial or supratentorial or middle cerebr$ or mca$ or anterior circulation) adj5 (isch?emi$ or infarct$ or thrombo$ or emboli$ or occlus$ or hypoxi$)).tw.
4. 1 or 2 or 3
5. exp Hypothermia,Induced/
6. exp Cryotherapy/
7. exp Body Temperature/
8. (hypotherm* or normotherm* or cool* or cold* or temperature* or cryother* or cryogen* or cryotreat*).ti,ab.
9. 5 or 6 or 7 or 8
10. 4 and 9
11. randomized controlled trial.pt.
12. controlled clinical trial.pt.
13. randomized.ab.
14. placebo.ab.
15. clinical trials as topic.sh.
16. randomly.ab.
17. trial.ti.
18. 11 or 12 or 13 or 14 or 15 or 16 or 17
19. exp animals/ not humans.sh.
20. 18 not 19
21. 10 and 20

Supplementary figures

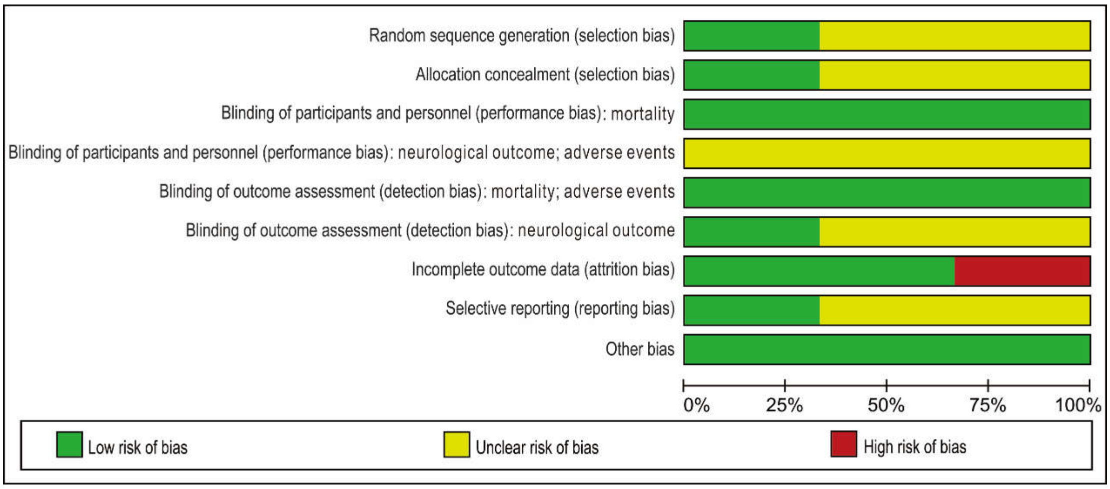

Figure S1: Risk of bias graph: review authors' judgements about each risk of bias itempresented as percentages across all included studies.

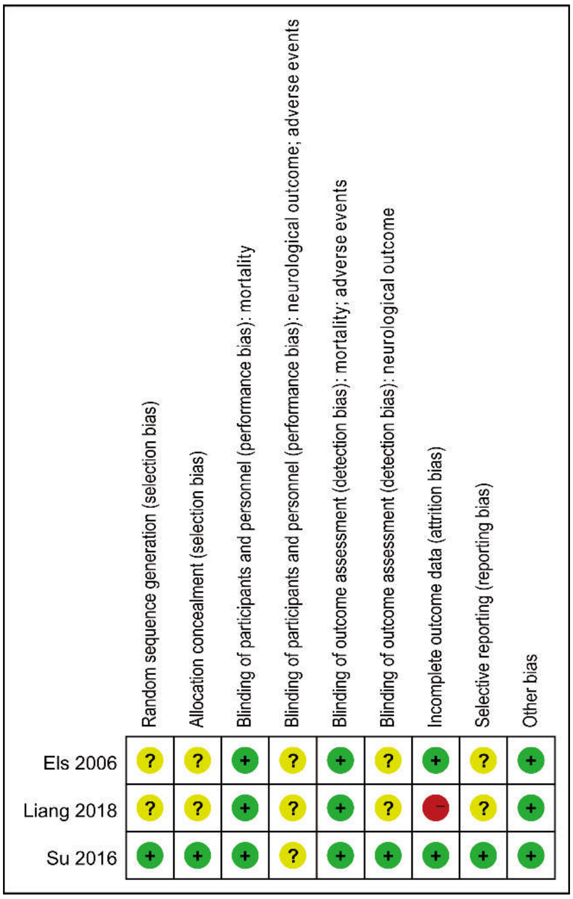

Figure S2:Risk of bias summary: review authors' judgements about each risk of bias item foreach included study.

Study or Subgroup	Hypothermia Events	Hypothermia Total	Control Events	Control Total	Weight	Risk Ratio M-H, Fixed, 95% CI
**1.5.1 Developed herniation in the rewarming process**						
Liang 2018	6	37	4	36	100.0%	1.46 [0.45, 4.74]
**Subtotal (95% CI)**		**37**		**36**	**100.0%**	**1.46 [0.45, 4.74]**
Total events	6		4			
Heterogeneity: Not applicable						
Test for overall effect: Z = 0.63 (P = 0.53)						
**1.5.2 Pneumonia**						
Liang 2018	18	37	10	36	56.6%	1.75 [0.94, 3.26]
Su 2016	7	16	8	17	43.4%	0.93 [0.44, 1.97]
**Subtotal (95% CI)**		**53**		**53**	**100.0%**	**1.40 [0.87, 2.25]**
Total events	25		18			
Heterogeneity: Chi² = 1.64, df = 1 (P = 0.20); I² = 39%						
Test for overall effect: Z = 1.37 (P = 0.17)						
**1.5.3 Cardiac arrhythmia**						
Liang 2018	21	37	15	36	83.9%	1.36 [0.84, 2.20]
Su 2016	1	16	3	17	16.1%	0.35 [0.04, 3.06]
**Subtotal (95% CI)**		**53**		**53**	**100.0%**	**1.20 [0.75, 1.91]**
Total events	22		18			
Heterogeneity: Chi² = 1.50, df = 1 (P = 0.22); I² = 33%						
Test for overall effect: Z = 0.77 (P = 0.44)						
**1.5.4 Hemorrhagic transformation**						
Liang 2018	3	37	4	36	67.6%	0.73 [0.18, 3.03]
Su 2016	1	16	2	17	32.4%	0.53 [0.05, 5.31]
**Subtotal (95% CI)**		**53**		**53**	**100.0%**	**0.67 [0.20, 2.23]**
Total events	4		6			
Heterogeneity: Chi² = 0.05, df = 1 (P = 0.82); I² = 0%						
Test for overall effect: Z = 0.66 (P = 0.51)						
**1.5.5 Gastrointestinal bleeding**						
Liang 2018	18	37	6	36	75.8%	2.92 [1.31, 6.51]
Su 2016	8	16	2	17	24.2%	4.25 [1.06, 17.08]
**Subtotal (95% CI)**		**53**		**53**	**100.0%**	**3.24 [1.62, 6.49]**
Total events	26		8			
Heterogeneity: Chi² = 0.21, df = 1 (P = 0.65); I² = 0%						
Test for overall effect: Z = 3.32 (P = 0.0009)						
**1.5.6 Gastric retention**						
Liang 2018	16	37	6	36	47.3%	2.59 [1.14, 5.88]
Su 2016	15	16	7	17	52.7%	2.28 [1.27, 4.07]
**Subtotal (95% CI)**		**53**		**53**	**100.0%**	**2.43 [1.47, 3.99]**
Total events	31		13			
Heterogeneity: Chi² = 0.07, df = 1 (P = 0.79); I² = 0%						
Test for overall effect: Z = 3.49 (P = 0.0005)						
**1.5.7 Hyperglycemia**						
Liang 2018	9	37	10	36	91.3%	0.88 [0.40, 1.90]
Su 2016	6	16	1	17	8.7%	6.38 [0.86, 47.29]
**Subtotal (95% CI)**		**53**		**53**	**100.0%**	**1.36 [0.68, 2.69]**
Total events	15		11			
Heterogeneity: Chi² = 3.51, df = 1 (P = 0.06); I² = 72%						
Test for overall effect: Z = 0.87 (P = 0.38)						
**1.5.8 Hypotension**						
Liang 2018	5	37	3	36	51.1%	1.62 [0.42, 6.29]
Su 2016	6	16	3	17	48.9%	2.13 [0.64, 7.10]
**Subtotal (95% CI)**		**53**		**53**	**100.0%**	**1.87 [0.76, 4.60]**
Total events	11		6			
Heterogeneity: Chi² = 0.09, df = 1 (P = 0.77); I² = 0%						
Test for overall effect: Z = 1.36 (P = 0.17)						
**1.5.9 Electrolyte derangement**						
Liang 2018	19	37	13	36	58.8%	1.42 [0.83, 2.43]
Su 2016	16	16	9	17	41.2%	1.84 [1.18, 2.87]
**Subtotal (95% CI)**		**53**		**53**	**100.0%**	**1.59 [1.12, 2.28]**
Total events	35		22			
Heterogeneity: Chi² = 0.57, df = 1 (P = 0.45); I² = 0%						
Test for overall effect: Z = 2.56 (P = 0.01)						
**1.5.10 Shivering**						
Liang 2018	18	37	3	36	100.0%	5.84 [1.88, 18.12]
**Subtotal (95% CI)**		**37**		**36**	**100.0%**	**5.84 [1.88, 18.12]**
Total events	18		3			
Heterogeneity: Not applicable						
Test for overall effect: Z = 3.05 (P = 0.002)						
**1.5.11 Acute kidney injury**						
Liang 2018	5	37	1	36	67.6%	4.86 [0.60, 39.63]
Su 2016	2	16	0	17	32.4%	5.29 [0.27, 102.49]
**Subtotal (95% CI)**		**53**		**53**	**100.0%**	**5.00 [0.90, 27.71]**
Total events	7		1			
Heterogeneity: Chi² = 0.00, df = 1 (P = 0.96); I² = 0%						
Test for overall effect: Z = 1.84 (P = 0.07)						
**1.5.12 Venous thrombotic events**						
Su 2016	4	16	2	17	100.0%	2.13 [0.45, 10.05]
**Subtotal (95% CI)**		**16**		**17**	**100.0%**	**2.13 [0.45, 10.05]**
Total events	4		2			
Heterogeneity: Not applicable						
Test for overall effect: Z = 0.95 (P = 0.34)						

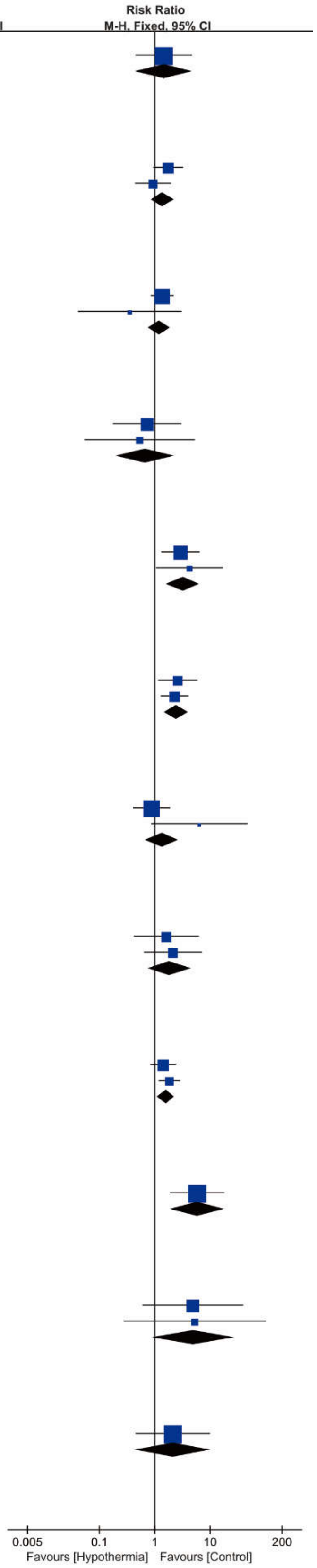

Figure S3: Meta-Analysis of association between hypothermia and the adverse events during treatment for LHI (including the result of hypothermia and hyperglycemia).

Study or Subgroup	Hypothermia Events	Hypothermia Total	Control Events	Control Total	Weight	Risk Ratio IV, Random, 95% CI
**1.5.1 Developed herniation in the rewarming process**						
Liang 2018	6	37	4	36		Not estimable
**Subtotal (95% CI)**		**0**		**0**		**Not estimable**
Total events	0		0			
Heterogeneity: Not applicable						
Test for overall effect: Not applicable						
**1.5.2 Pneumonia**						
Liang 2018	18	37	10	36		Not estimable
Su 2016	7	16	8	17		Not estimable
**Subtotal (95% CI)**		**0**		**0**		**Not estimable**
Total events	0		0			
Heterogeneity: Not applicable						
Test for overall effect: Not applicable						
**1.5.3 Cardiac arrhythmia**						
Liang 2018	21	37	15	36		Not estimable
Su 2016	1	16	3	17		Not estimable
**Subtotal (95% CI)**		**0**		**0**		**Not estimable**
Total events	0		0			
Heterogeneity: Not applicable						
Test for overall effect: Not applicable						
**1.5.4 Hemorrhagic transformation**						
Liang 2018	3	37	4	36		Not estimable
Su 2016	1	16	2	17		Not estimable
**Subtotal (95% CI)**		**0**		**0**		**Not estimable**
Total events	0		0			
Heterogeneity: Not applicable						
Test for overall effect: Not applicable						
**1.5.5 Gastrointestinal bleeding**						
Liang 2018	18	37	6	36		Not estimable
Su 2016	8	16	2	17		Not estimable
**Subtotal (95% CI)**		**0**		**0**		**Not estimable**
Total events	0		0			
Heterogeneity: Not applicable						
Test for overall effect: Not applicable						
**1.5.6 Gastric retention**						
Liang 2018	16	37	6	36		Not estimable
Su 2016	15	16	7	17		Not estimable
**Subtotal (95% CI)**		**0**		**0**		**Not estimable**
Total events	0		0			
Heterogeneity: Not applicable						
Test for overall effect: Not applicable						
**1.5.7 Hyperglycemia**						
Liang 2018	9	37	10	36	61.3%	0.88 [0.40, 1.90]
Su 2016	6	16	1	17	38.7%	6.38 [0.86, 47.29]
**Subtotal (95% CI)**		**53**		**53**	**100.0%**	**1.89 [0.28, 12.57]**
Total events	15		11			
Heterogeneity: Tau² = 1.37; Chi² = 3.28, df = 1 (P = 0.07); I² = 70%						
Test for overall effect: Z = 0.66 (P = 0.51)						
**1.5.8 Hypotension**						
Liang 2018	5	37	3	36		Not estimable
Su 2016	6	16	3	17		Not estimable
**Subtotal (95% CI)**		**0**		**0**		**Not estimable**
Total events	0		0			
Heterogeneity: Not applicable						
Test for overall effect: Not applicable						
**1.5.9 Electrolyte derangement**						
Liang 2018	19	37	13	36		Not estimable
Su 2016	16	16	9	17		Not estimable
**Subtotal (95% CI)**		**0**		**0**		**Not estimable**
Total events	0		0			
Heterogeneity: Not applicable						
Test for overall effect: Not applicable						
**1.5.10 Shivering**						
Liang 2018	18	37	3	36		Not estimable
**Subtotal (95% CI)**		**0**		**0**		**Not estimable**
Total events	0		0			
Heterogeneity: Not applicable						
Test for overall effect: Not applicable						
**1.5.11 Acute kidney injury**						
Liang 2018	5	37	1	36		Not estimable
Su 2016	2	16	0	17		Not estimable
**Subtotal (95% CI)**		**0**		**0**		**Not estimable**
Total events	0		0			
Heterogeneity: Not applicable						
Test for overall effect: Not applicable						
**1.5.12 Venous thrombotic events**						
Su 2016	4	16	2	17		Not estimable
**Subtotal (95% CI)**		**0**		**0**		**Not estimable**
Total events	0		0			
Heterogeneity: Not applicable						
Test for overall effect: Not applicable						

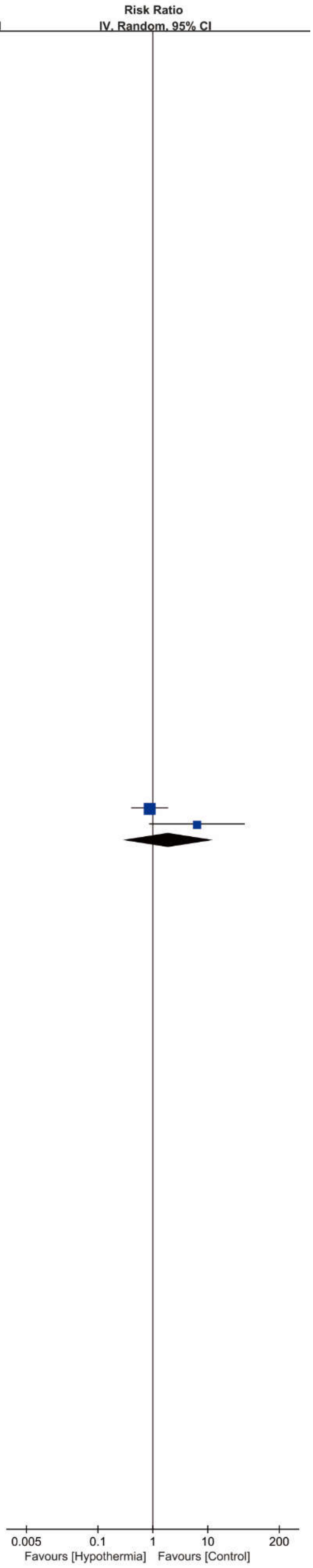

Figure S4: Meta-Analysis of association between hypothermia and hyperglycemia for LHI (a random effects model was applied here).
